# Anti-Coccal Activity and Composition of the Essential Oils and Methanolic Extracts Obtained from Brewing Quality *Humulus lupulus* L. Hop Pellets

**DOI:** 10.3390/ph16081098

**Published:** 2023-08-03

**Authors:** Bartłomiej Piasecki, Anna Biernasiuk, Agnieszka Ludwiczuk

**Affiliations:** 1 Department of Pharmacognosy with the Medicinal Plant Garden, Medical University of Lublin, 1 Chodźki Str., 20-093 Lublin, Poland; 2 Department of Pharmaceutical Microbiology, Medical University of Lublin, 1 Chodźki Str., 20-093 Lublin, Poland; annabiernasiuk@umlub.pl

**Keywords:** hop cultivars, antibacterial, extract, essential oil, xanthohumol

## Abstract

This study examined the chemical composition and anti-coccal properties of essential oils and methanolic extracts of six different *Humulus lupulus* L. varieties from Poland: Iunga, Marynka, Sybilla, Magnum, Tradition and Chinook. The activity of an α-acid-enriched fraction of methanolic extracts was also studied. The chemical composition of essential oils and extracts was determined by gas chromatography–mass spectrometry (GC/MS) and liquid chromatography–mass spectrometry (LC/MS) techniques. The compounds characteristic to *H. lupulus* extracts include xanthohumol, α-acids, β-acids, and prenylated flavonoids. Essential oil compositions showed a high prevalence of monoterpene hydrocarbon, myrcene and sesquiterpene hydrocarbons, α-humulene and β-caryophyllene. The antimicrobial activity was investigated against eight human cocci pathogenic strains: *Staphylococcus aureus* MRSA (ATCC 43300), *S. aureus* MRSA (29213), *S. aureus* MSSA (ATCC 29213), *S. epidermidis* (ATCC 12228), *Enterococcus faecalis* (ATCC 29212), *E. faecalis* VRE (ATCC 51299), *E. faecium* (ATCC 19434) and *Micrococcus luteus* (ATCC 10240). The lowest minimum inhibitory concentrations (MIC) were obtained for extracts and essential oils from Iunga hop samples. Extracts were significantly more active than essential oils. The most susceptible strain to both essential oils and extracts was *M. luteus*, whilst the least susceptible was *E. faecium*. The antimicrobial activity correlated with a high concentration of xanthohumol of active extracts rather than with the content of α-acids. Xanthohumol showed considerable activity against MRSA with an MIC value of 3.9 µg/mL. The activity of the α-acid-enriched fraction was mediocre compared to the results of all extracts.

## 1. Introduction

*Humulus lupulus* L. (hop) is a perennial, dioecious herb from the Cannabaceae family. The plant grows in the form of a vine which dies at the end of the season and recreates from rhizomes in the spring [1]. Cone-like female flowers called ‘hop cones’ are the organs with the highest concentration of desired compounds like bitter acids and volatile terpenoids, both being the main reason for cultivation for brewing purposes. Male plants are not used in the brewing industry as they do not produce bitter agents, but they are necessary in producing new varieties and are therefore cultivated as well. The main application for hops lies in the brewing industry, but additionally, extracts can be used in cosmetics and medicine due to natural products with multiple properties. The brewing industry, as well as pharmaceutical and cosmetic industries, are switching from using raw hop material to hop extracts (supercritical CO_2_ or ethanol) in order to obtain a more reliable and consistent source with a known content of desired compounds [2].

The phytochemistry of *H. lupulus* is complex due to the existence of many wild and cultivated varieties. This complex chemical composition makes hops an interesting source of bioactive compounds and thus a variety of pharmaceutical and medical applications are described. An aqueous infusion of hop cones is known for its sedative properties and extracts can exhibit antiseizure activity [3,4,5,6]. Other research has focused on the antimicrobial properties of hops and there is strong and substantial evidence of its activity not only against bacterial strains but also against fungi and viruses [7,8,9,10]. 

The most important group of compounds present in *H. lupulus* are α-acids and their isomers, which are phloroglucinol derivatives that impart the strong, bitter taste of beer. Isomerization is an important reaction due to the stronger bitterness of the iso-α acids compared to α-acids. This reaction takes place during boiling the wort at a temperature of approximately 100 °C and an acidic pH [2]. 

**Figure 1:**
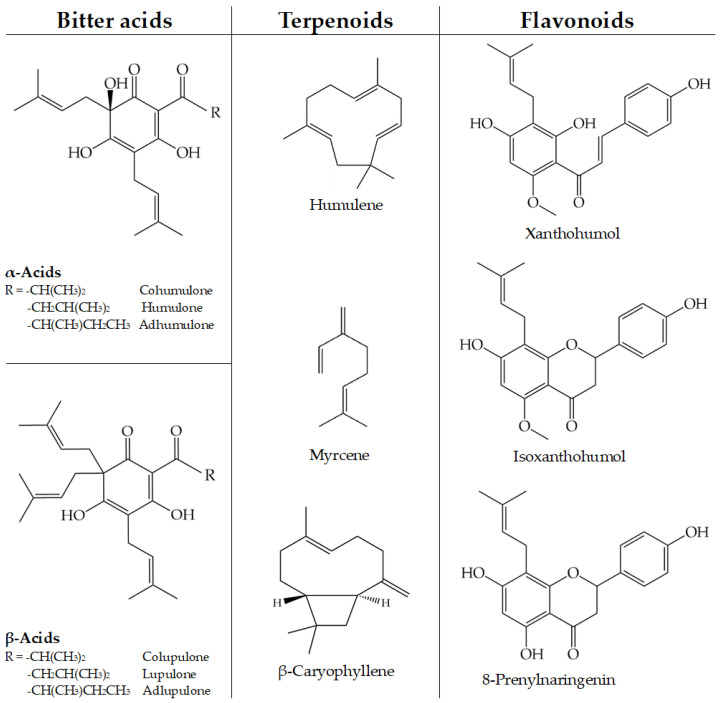
The important phytochemical groups found in *Humulus lupulus* L. with their most abundant representatives. Based on the data from [2].

Due to different concentrations of bittering agents in the raw material, hops can be divided into bitter hops with a high content of α-acids, aroma hops with a lower content of α-acids but higher yield of essential oils (EOs), and hops that share both groups and their characteristics. Bitter hops usually have an α-acid content up to 18%, while this content in aroma hops do not exceed 6%. The composition of EOs obtained from aroma hops is dominated by the presence of representatives of terpene hydrocarbons, namely myrcene, α-humulene and β-caryophyllene [2,11]. α-acids are represented by five compounds: humulone, cohumulone, adhumulone, prehumulone and posthumulone, but pre- and posthumulone occur at very low concentrations. All α-acids have their corresponding β-acids: lupulone, colupulone, adlupulone and prelupulone, but they lack bittering properties due to their structural differences and low water solubility. However, they do participate in the antimicrobial activity of hops [12]. *H. lupulus* is also a source of prenylated chalcones and flavonoids such as xanthohumol, isoxanthohumol and 8-prenylnaringenin. Although being minor constituents, they have strong antibacterial properties (xanthohumol) or modulate female hormone activity (8-prenylnaringenin) [13]. Some of the most prevalent hops compounds are shown in Figure 1. 

Many human pathogens are bacteria of the cocci group, which cause some severe infections, for example, *Staphylococcus aureus* (inflammatory, skin infections, food poisoning, pneumonia), *Streptococcus pneumoniae* (pneumonia, sinus infections and meningitis) and *Enterococcus* (colon and urinary tract infections) [14]. There are no tissues within the human body that cannot be infected by these microorganisms and their antibiotic resistance is causing problems every year [15]. Due to the decreasing activity of antibiotics against some of these species, there is a pressing need for new antibacterials and, therefore, plant-based therapeutics are a popular solution [16]. 

For this purpose, EOs and methanolic extracts from six different hop cultivars from Poland were obtained. The aim of this study was the correlation of anti-coccal activity of EOs and extracts with their chemical composition. To the best of our knowledge, this is the first study to determine the antimicrobial activity of hop EOs and extracts against eight human cocci pathogenic strains, and to relate this activity with their phytochemistry (volatile and non-volatile compounds) in order to establish which cultivars and compounds have the best potential. 

## 2. Results

### 2.1. Phytochemical Evaluation of Hop Cultivars

Six hop varieties cultivated in Poland were hydro-distilled to afford essential oils (EOs) and extracted with methanol. The results of the EO content and extraction efficiency are presented in Table 1. 

The content of EOs ranged from 0.6 to 1.5%. The highest EO content was characteristic for samples of Magnum and Marynka hops with a yield of 1.5 and 1.3%, respectively. The lowest EO content was associated with the Tradition and Chinook cultivars with 0.6 and 0.68%, respectively. The extraction of hop samples with methanol as a solvent showed the highest efficiency for Iunga (6.98%), Magnum (6.92%) and Tradition (6.28%) cultivars. The lowest extraction efficiency was from the Sybilla hop sample (5.32%). The differences in the EO content were far more significant than the extraction efficiency. 

In all MeOH extracts, the concentration of xanthohumol was determined by HPLC. As shown in Table 1, the highest content of this compound was found in an extract from the Iunga cultivar (2.71%). In this case, the high amount of xanthohumol was correlated with high extraction efficiency. On the other hand, the MeOH extract from Magnum hop had the lowest content of this compound (0.39%), but the extraction efficiency for this cultivar was comparable to Iunga hop.

The composition of volatile components in all obtained EOs and MeOH extracts were determined by GC/MS. The non-volatiles present in methanolic extracts were analyzed by LC/MS. 

The GC/MS results for the EOs are shown in Table 2. The major volatile component present in all analyzed EOs is the monoterpene hydrocarbon, myrcene. Its relative percentage ranged from 24% in Tradition (Tr5) and Chinook (Ch6) hop EOs to 37% in the Magnum (Mg4) cultivar.

Besides myrcene, the EOs showed a high prevalence of two more hydrocarbons but from the sesquiterpene class. These were α-humulene and (*E*)-β-caryophyllene. Based on the relative percentages of these two compounds, the investigated hop cultivars could be divided into two groups. In the first, the relative percentage of the mentioned sesquiterpene hydrocarbons were above 40%. The EOs with such a content of these compounds were Iunga (In1) and Tradition (Tr5). The relative percentage of α-humulene and (*E*)-β-caryophyllene in the remaining four EOs was between 21% and 35%. The essential oil obtained from the Marynka variety was the only sample containing (*E*)-β-farnesene as the second most abundant compound. This compound represented 18.8% of all compounds present in this EO. The relative percentage of (*E*)-β-farnesene in the other EOs was from 0.9% for In1 (Iunga) and 8.1% for Sb3 (Sybilla).

The most prevalent oxygenated compounds were α-humulene epoxide II and esters but none of these exceeded 3% of the identified compounds. 

GC/MS analysis of the volatile compounds present in the methanolic extracts obtained from all investigated hop samples confirmed the presence of α-humulene, (*E*)-β-caryophyllene and (*E*)-β-farnesene (Table 3). As shown in Table 3, in addition to the mentioned sesquiterpenes, among the volatile components present in hop extracts, the β-acids colupulone, adlupulone, and lupulone were also identified. Due to the same molecular mass and very similar fragmentation pattern, a distinction between adlupulone and lupulone was possible, as the latter occurs in higher quantities in hops.

All identified compounds are characteristic of hops extracts in general, although under the conditions of this GC/MS separation, the α-acids, because of low volatility, were either not detected or in negligibly small amounts and therefore not present in the table.

For a better characterization of the chemical composition of the methanolic extracts, an LC-MS analysis was also performed. A liquid chromatograph coupled with a QTOF spectrometer was used for this purpose. A high mass accuracy, characteristic for this type of spectrometer, allows the determination, with a very high probability, of the molecular formulae for the analyzed compounds. This high accuracy of the measured mass means that usually only a few of these formulae are generated, which facilitates identification. The results from the HPLC/ESI-QTOF-MS in a negative ion mode are shown in Table 4.

Among identified compounds, all are characteristic of hops extracts. The α-acids were represented by humulone, cohumulone and adhumulone. In contrast to the GC/MS results, α-acids were the abundant compounds with very low to no identified β-acids in the negative ion mode. 

Another characteristic group of compounds detected in all analyzed extracts were the prenylflavonoids. These were represented by the prenylated chalcone, xanthohumol, as well as by prenylated flavanones. The presence of 8-prenylnaringenin and naringenin glucuronide was confirmed. Additionally, an unidentified flavonoid with a molecular mass of 444 Da was also present.

### 2.2. Anti-Coccal Activity of Hop Essential Oils and Extracts

The antimicrobial activity of EOs and methanolic extracts, as well as an α-acid-enriched fraction (α-AEF) was investigated against eight reference human cocci pathogenic strains. These were methicillin-resistant strains *Staphylococcus aureus* MRSA (ATCC 43300) and *S. aureus* MRSA (29213); methicillin-sensitive strain *S. aureus* MSSA (ATCC 29213), *S. epidermidis* (ATCC 12228), *Enterococcus faecalis* (ATCC 29212), vancomycin-resistant strain *E. faecalis* VRE (ATCC 51299), *E. faecium* (ATCC 19434), and *Micrococcus luteus* (ATCC 10240). Additionally, the antimicrobial activity of xanthohumol against *S. aureus* MRSA (ATCC 43300) was examined.

#### 2.2.1. Essential Oils

The results of the minimum inhibitory concentration (MIC) and minimum bactericidal concentration (MBC) assays for the EOs are shown in Table 5. These data show that the studied EOs had some antimicrobial effects against all reference Gram-positive bacteria, with MIC values ranging from 1 to 16 mg/mL, and MBC values from 2 to 32 mg/mL.

The lowest MICs were from the EO of Iunga (In1) and Chinook (Ch6) against *Micrococcus luteus* (1 mg/mL). A similar effect of Tradition EO (Tr5) towards the strains of *S. epidermidis* and *M. luteus* was also observed (MIC = 2 mg/mL). The similar antimicrobial activity of EOs from Iunga and Tradition cultivars could be explained by similar chemical composition of EOs. Both were characterized by a high concentration of α-humulene and (*E*)-β-caryophyllene. The least-susceptible species were *E. faecalis* (ATCC 29212) and *E. faecium* (ATCC 19434), where all EOs showed MIC values of 16 mg/mL, except for the Iunga cultivar with 8 mg/mL. The latter two strains also showed the highest MBC values, which did not go below 32 mg/mL. 

#### 2.2.2. Methanol Extracts and the α-Acid-Enriched Fraction (α-AEF)

The results of the MIC and MBC assays for the methanol extracts and the α-AEF are shown in Table 6. All extracts and α-AEF showed appreciable antimicrobial activity against the selected reference cocci belonging to Gram-positive bacteria with MIC values ranging from 7.8 µg/mL to 62.5 µg/mL (MBC = 7.8–>2000 µg/mL). These data prove that the activity of the hop extracts is at least one order of magnitude greater than that of essential oils, shown in Table 5.

The microorganisms were most sensitive to the extracts of Iunga (In1), Sybilla (Sb3) and Tradition (Tr5), which exhibited antibacterial effects with MIC values between 7.8–31.3 µg/mL and MBC values between 7.8–2000 µg/mL. In the case of the Iunga hop extract, activity was high (MIC = 7.8 µg/mL) for all reference strains of *S. aureus* (both MRSA and MSSA), *M. luteus*, *E. faecalis* ATCC 29212, and *E. faecium* ATCC 19434. The same effect was observed for the Sybilla hop extract against the two strains, *S. aureus* MRSA and *M. luteus*, and the extract of Tradition cultivar against one of the staphylococci (*S. aureus* ATCC 43300) and *M. luteus*. The activity of the Iunga, Sybilla and Tradition extracts against the other bacteria was also strong (MIC = 15.6 µg/mL) or good (MIC = 31.3 µg/mL). In the case of the Marynka (Mr2) and Chinook (Ch6) extracts, activity was similar—strong or good with MIC values in the range from 15.6 µg/mL to 62.5 µg/mL (MBC = 15.6–2000 µg/mL). In turn, the antibacterial effect of Magnum (Mg4) hop extract was slightly lower with good activity (MIC = 31.3–62.5 µg/mL). Moreover, α-AEF showed similar effects, with MIC values ranging from 15.6 to 62.5 µg/mL. It should be added that these extracts showed appreciable bactericidal activity (with some exceptions) towards staphylococci and micrococci (MBC/MIC = 1–4) and bacteriostatic effects against the enterococci (MBC/MIC = 8–>64).

Very strong anti-coccal activity of the extract obtained from the Iunga hop cultivar (In1) could be correlated with the highest concentration of xanthohumol in this extract (see Table 1). To prove this, the antimicrobial activity of xanthohumol against the *S. aureus* MRSA (ATCC 43300) strain was determined. The results showed that xanthohumol activity was also potent, with an MIC value of 3.9 µg/mL. In comparison with the Iunga hop extract, the MBC value of xanthohumol was 250 µg/mL and the MBC/MIC ratio was 64. The MBC/MIC ratio indicated bacteriostatic properties of xanthohumol against this particular MRSA strain. Xanthohumol was significantly more active against the MRSA strain (ATCC 43300) than the α-AEF prepared in this study, but the latter, although exhibiting higher MIC values, showed bactericidal properties.

## 3. Discussion

The antimicrobial properties of hops have made it a popular additive material in beer for centuries [17]. With the development of a scientific approach to plant-based therapeutics, hops have found their way into the scope of microbiological studies. Different classes of chemicals found in hops extracts exert significant antimicrobial properties: prenylated chalcones and flavonoids, mono- and sesquiterpenes, and phloroglucinol derivatives [18]. 

The antimicrobial activity of hop EOs has been the subject of studies. Jirovetz et al. [19] investigated the antimicrobial activity and chemical composition of EOs obtained from Bavarian hop. The most characteristic components of this oil were myrcene, α-humulene, and (*E*)-β-caryophyllene, and it was active against the Gram-positive bacteria *S. aureus* and *E. faecalis* with an MIC value of 600 µg/mL. The authors also examined the major and minor compounds of the EO and concluded that activity did not come from a single compound. Langezaal and coworkers [7] examined the antimicrobial activities of EOs and chloroform extracts of the corresponding hop samples. They used an agar overlay assay method to establish and compare inhibition zones. Their results showed that extracts were significantly more active than EOs and this is in agreement with the results of our work. 

Schmalreck et al. [20] tested bitter resin extracts of hops against *B. subtilis* and concluded that the prenyl groups, responsible for hydrophobic properties, are indeed an important factor for interacting with the bacterial cell wall. This mechanism would suggest a higher activity for β-acids (three prenyl chains, Figure 1) compared to the α-acids (two prenyl chains); therefore, hop extracts with a higher β-acid content should have better antimicrobial properties [20]. Sawicka et al. [21] concluded that the content of the α-acids and β-acids in hops depends on variety, location, and meteorological conditions. In our study, it was not possible to readily connect the anti-coccal properties of the hop extracts with α-acid content. The results of the α-AEF showed that the anti-coccal activity of this fraction was average compared to all tested hop extracts. Meanwhile, comparing the activity of extracts and xanthohumol against an MRSA (ATCC 43300) strain, it was observed that the MIC value for xanthohumol was twice lower than the lowest results for the extracts obtained from the most active hops. Xanthohumol activity could be correlated with its content in the investigated extracts. Hop methanol extracts with the highest xanthohumol concentration were Iunga with 2.69% and Sybilla with 1.80%, and these extracts showed the highest anti-coccal activity. On the other hand, an extract obtained from the Magnum hop variety showed the lowest xanthohumol concentration (0.39%) and the lowest antimicrobial activity. These results supported the xanthohumol-dependent antimicrobial activity of hop extracts. 

There are also some examples from the literature that are in agreement with our results. Similar studies were conducted by Różalski et al. [22] using the dilution method against three strains of *S. aureus* of different origin. An extract of hop cones containing 51% xanthohumol was slightly less active against *S. aureus* strains (MIC = 31.2–125 μg/mL and MBC = 31.2–500 μg/mL) than pure xanthohumol (MIC = 15.6–62.5 μg/mL and MBC = 250–>500 μg/mL). On the other hand, the spent hop extract, free of xanthohumol, exhibited lower but still relevant activity (MIC = 1–2 mg/mL and MBC ≥ 2 mg/mL). In the case of *E. faecalis*, activity of an extract of hop cones and pure xanthohumol was the same—62.5 μg/mL—while MBC values were 1 mg/mL and >500 μg/mL, respectively. In turn, the spent hop extract showed activity at an MIC = 1–2 mg/mL and MBC ≥ 2 mg/mL [22].

In the study of Bogdanova et al. [9] the antibacterial activities of xanthohumol against different *Staphylococcus* species, including strains causing life-threatening biofilm-associated infections of artificial heart valves, were assessed. Methicillin-susceptible *Staphylococcus epidermidis* and *S. aureus* strains, three methicillin-resistant strains, namely *S. epidermidis*, *S. capitis*, as well as *S. aureus,* which had been isolated from an intravenous catheter of a diseased patient, were included. The analyses revealed MICs of xanthohumol of <4 μg/mL, and an MBC ranging from 1 to 15 μg/mL, against all tested strains. Additionally, xanthohumol was able to penetrate the biofilm and reduce the number of bacteria within it at a concentration of 60 μg/mL. This study showed potent anti-staphylococcal and biofilm-reducing effects of xanthohumol [9].

In order to test the antibacterial activity of xanthohumol against *S. aureus* and *S. epidermidis*, Bartmanska et al. [23] used spent hops. The content of xanthohumol and its derivatives ranged from 0.1 to 1% of the cone dry mass. The antibacterial effect was assessed by measurements of the MIC_80_ (defined as the concentration inhibiting 80% of the bacterial growth). The analyses revealed that xanthohumol and its derivatives showed activity against methicillin-sensitive and resistant strains of *S. aureus* and *S. epidermidis,* with MIC_80_ values ranging from 5 to 50 μg/mL, whereas using ampicillin as the antibacterial control yielded MIC_80_ values between 2.5 and 5 μg/mL, respectively. The authors suggested further investigations addressing some compounds from spent hops and their derivatives as potential treatment options of staphylococcal infections [23]. 

According to data from Cheng et al. [24], one of the novel amphiphilic xanthohumol derivative exhibited a remarkable antibacterial effect against clinical MRSA isolates (MIC in the range 1–2 μg/mL). The tested compound has a good membrane-targeting ability and can bind to phosphatidylglycerol and cardiolipin in bacterial membranes, thus disrupting the bacterial cell membranes and causing increased intracellular reactive oxygen species and the leakage of proteins and DNA, eventually resulting in bacterial death [24]. 

In turn, Cermak et al. [25] determined the antimicrobial activity of xanthohumol against anaerobic bacteria: *Bacteroides fragilis*, *Clostridium perfringens* and *Clostridioides difficile* strains. The lowest MICs and thus the most effective antibacterial effect were obtained for xanthohumol ranging from 10 to 56 μg/mL in the case of *B. fragilis* and *C. perfringens*, respectively, whereas the MBCs were slightly higher (up to 80 μg/mL). The antibacterial activity against *C. difficile* was observed at an MIC from 32 μg/mL to 107 μg/mL and an MBC between 40 μg/mL and 107  μg/mL, which are close to those of conventional antibiotics in the strains of bacteria with increased resistance. The authors concluded that these results were close to the MICs and MBCs when testing conventional synthetic antibiotics against respective bacterial isolates, indicating the potent antibacterial effects of defined hop ingredients against anaerobic pathogens [25]. Sleha et al. [12] also investigated the activity of different hop-derived compounds against *Clostridioides difficile*, and found xanthohumol to be the most active antibacterial agent with anti-inflammatory properties as well. The next studies [26] showed that xanthohumol demonstrated antimicrobial activity against the six other bacterial species growing on the surfaces of dental implants (*Streptococcus oralis*, *Veillonella parvula*, *Actinomyces naeslundii*, *Fusobacterium nucleatum*, *Porphyromonas gingivalis* and *Aggregatibacter actinomycetemcomitans*) at an MIC and MBC in the range of 10–100 μM. 

The activity of hop extract has also been studied by other authors. Weber et al. [27] showed inhibition of the growth of MRSA at 12.5 μg/mL, whereas the clindamycin MIC was 50 μg/mL. The experiments conducted by Kramer et al. [28] exhibited strong antibacterial activity of hop extracts containing β-acids and xanthohumol against *Staphylococcus aureus*, *Listeria monocytogenes* and other Gram-positive bacteria, as well as acid-fast bacteria and fungi (MIC values of 6.3 and 12.5 ppm, respectively), whereas the antimicrobial activity of the investigated α-acid extract was significantly lower (MIC values of 200 ppm). Gram-negative bacteria were highly resistant against all tested hop extracts. Their results indicated that hop extracts can be used as natural food preservatives. Kolenc et al. [29] compared the susceptibility of two strains of *Lactobacillus acidophilus* (ATCC 4356) and MSSA *Staphylococcus aureus* (ATCC 29213) to different hydroacetonic hops extracts. Their findings gave comparable results for the strain (MSSA) used in our work and gave MICs values in the range of 9.8 to 54.7 µg/mL. The authors concluded that xanthohumol and the bitter acids are responsible for the antimicrobial properties of all hop samples [29]. 

Depending on the method of the extraction, hop extracts can either contain a high percentage or only trace amounts of xanthohumol. This is the case of supercritical fluid extraction (SFE). Recently, many industries (brewing, pharmaceutical, cosmetic) are switching from using raw hop materials to hop extracts, and supercritical CO_2_ extracts are extremely popular. In a study conducted by Klimek et al. [30], a two-step supercritical fluid extraction (SFE) was applied in order to obtain two hop samples. The first one, called “crude extract” contained humulone, lupulone, terpenoids and xanthohumol, whereas the second extract was pure xanthohumol. Both extracts were tested against *S. aureus*, *S. epidermidis*, *Streptococcus mutans*, *S. sanguinis* and *Propionibacterium acnes*. Both extracts were shown to be very effective against *S. epidermidis*, with MICs of 0.098 μg/mL. Similar antimicrobial activities were obtained for the synthetic antibiotics gentamicin and sparfloxacin against *S. epidermidis*. Slightly higher MICs values were measured against *S. aureus* (0.195 μg/mL) and *S. mutans* (0.391 μg/mL). Xanthohumol did not exert potent growth inhibition of *S. sanguinis*, but the “crude extract” inhibited the growth at an MIC of 0.781 μg/mL, similar to ciprofloxacin and even more effective than the third-generation cephalosporine ceftriaxone. Moreover, the hop extracts displayed relatively high MICs of 15.6–62.5 μg/mL against *P. acnes* strains [30]. 

Based on the literature data and our study, hop products, mainly those rich in xanthohumol, constituting a valuable source, and either alone or in combination with synthetic antibiotics might be considered promising options to combat infection caused by human pathogens. However, a lot more research, both in vitro and especially in vivo, is needed to provide more evidence for a translational application from bench to bedside.

## 4. Materials and Methods

### 4.1. Hop Samples

Six different *Humulus lupulus* L. varieties were examined: Iunga, Marynka, Sybilla, Magnum, Tradition and Chinook. All samples were purchased from www.homebeer.pl (accessed on 20 April 2022) in the form of pellets in tightly sealed packages. According to the seller, the hop cones came from various producers/farmers from the area in the Lublin Voivodeship, around the Vistula river. All varieties were cultivated in Poland, although only Iunga, Marynka and Sybilla are species native to Poland. The origin of the hop seedlings: National Research Institute in Puławy, Department of Plant Breeding and Biotechnology, Poland.

### 4.2. Essential Oil Hydro-Distillation

EOs were obtained by the hydro-distillation of 10 g hop pellets for 2 h in a Deryng-type apparatus. The volume of each sample was measured and the EO content in the hop material was calculated and shown in Table 1. The samples of the obtained EOs were stored in tightly sealed 1.5 mL amber vials at 4 °C prior to analysis.

### 4.3. Hop Extract Preparation

Extraction was conducted on material ground in a mortar. A weight of 50 g of each hops sample was extracted by an ultrasound-assisted extraction method with 50 mL of methanol twice for 20 min, and the third extraction was combined with maceration for 24 h. The mixture was then filtered, and methanol was evaporated under reduced pressure and a temperature of 35 °C. The masses of dry extracts were measured and the efficiency of extraction was calculated (Table 1). Each extract was kept in tightly sealed flasks at 4 °C prior to analysis. 

### 4.4. Acquisition of α-Acid-Enriched Fraction (α-AEF)

In order to investigate the impact of the α-acids on the overall antimicrobial activity of the methanol extracts, fractions enriched with these compounds were prepared. A specific reaction of α-acids with a methanolic solution of lead (II) acetate has been described in the literature [2,31,32,33]. The precipitate was washed with distilled water and centrifuged and dissolved in sulfuric acid. This dissolved acidic aqueous fraction was then extracted in a separating funnel with hexane. After evaporation of hexane, the mass of the obtained α-acid-enriched fraction was measured and a stock solution was prepared and used in the antimicrobial assay.

### 4.5. GC/MS Analysis of Volatile Compounds

Analyses were performed with a Shimadzu GC-2010 Plus instrument coupled to a Shimadzu QP2010 Ultra mass spectrometer (Shim-pol, Warsaw, Poland). Compounds were separated on a fused-silica capillary column ZB-5 MS (30 m, 0.25 mm i.d.) with a film thickness of 0.25 mm (Phenomenex, Torrance, CA, USA). The following oven temperature program was initiated at 50 °C, held for 3 min, then increased at a rate of 8–250 °C/min, and held for a further 2 min. The spectrometers were operated in EI mode; the scan range was 40–500 amu, the ionization energy 70 eV, and the scan rate was 0.20 s per scan. The injector, interface, and ion source were kept at 250, 250, and 220 °C, respectively. Split injection was conducted with a split ratio of 1:20, and helium was used as the carrier gas at a 1.0 mL/min flow rate. 

Operating conditions for the extracts were the same as described in the case of the EO except for the following: the oven temperature program was initiated at 50 °C, held for 3 min, then increased at a rate of 5–250 °C/min, and held for a further 15 min. Each of the six EO samples were prepared by diluting 2 µL of EO in 998 µL of hexane and in the case of the extracts, 20 mg of each sample was diluted in 1 mL of methanol. An internal standard was added to each sample. Three parallel measurements were made. The relative percentages of each component present in the analyzed samples were calculated. The retention indices were determined in relation to a homologous series of *n*-alkanes (C8–C28) under the same operating conditions. Compounds were identified using computer-assisted spectral libraries (MassFinder 2.1, Hamburg, Germany; NIST 2011, Gaithersburg, MD, USA). 

### 4.6. LC/MS Analysis of Methanolic Extracts

Samples of the methanol extracts were diluted with methanol to 10 mg/mL and analyzed qualitatively by an HPLC/ESI-QTOF-MS system in negative ion mode with the use of a 6530B Accurate-mass-QTOF-MS (Agilent Technologies, Inc., Santa Clara, CA, USA) mass spectrometer with an ESI-Jet Stream ion source. An Agilent 1260 chromatograph was equipped with a DAD detector, autosampler, binary gradient pump, and column oven. Gradient of solvents: water with 0.1% formic acid (solvent A) and acetonitrile with 0.1% formic acid (solvent B) were used as the mobile phases. The following gradient procedure was adopted: 0–45 min, 0–60% of B; 45–46 min, 60–95% B; 46–55 min 95% (B); the post time was 10 min. The total time of analysis was 65min, with a stable flow rate at 0.200 mL/min. The injection volume for extracts was 10 μL. ESI-QToF-MS analysis was performed according to the following parameters of the ion source: dual-spray jet stream ESI, positive and negative ion mode, gas (N2) flow rate: 12 L/min, nebulizer pressure: 35 psi, vaporizer temp: 300 °C; m/z range: 100–1000 mass units, with acquisition Mode Auto MS/MS, collision-induced dissociation (CID): 10 and 30 eV with MS scan rate 1 spectrum per s, 2 spectra per cycle, skimmer: 65 V, fragmentor: 140 V and octopole RF Peak: 750 V. The column used for this LC was a Phenomenex Gemini C18 column (2 × 100 mm, 3 μm, Torrance, CA, USA). The identification of major compounds was proceeded using Internet databases and in the case of xanthohumol by using a reference compound.

### 4.7. Quantitative Analysis of Xanthohumol by HPLC

A Shimadzu 20A series HPLC system (Shimadzu, Tokyo, Japan) coupled with an automatic degasser (DGU-20A 3R), quaternary pump (LC-20AD), auto-sampler (SIL-20 HT) and diode array detector (SPD-M20A) was used for the HPLC-DAD analyses. The separations were performed on the Agilent Zorbax Eclipse XDB-C18 (3.5 µm, 4.6 × 150 mm, Santa Clara, CA, USA) column. The conditions for the HPLC were as follows: gradient of solvents: water with 0.1% formic acid (solvent A) and acetonitrile with 0.1% formic acid (solvent B) were used as the mobile phases. The following gradient procedure was adopted: 0–45 min, 15% of B; 45–50 min, 70–95% B; 50–60 min 15% (B). The total time of analysis was 60 min with a stable flow rate at 1 mL/min. The injection volume for extracts was 10 μL. The concentration of xanthohumol present in the samples was determined from the response factors at 370 nm using a five-point calibration curve obtained with xanthohumol standard (Sigma-Aldrich, Merck KGaA, Darmstadt, Germany) (5–250 μg/mL, y = a × 5.19E + 12, R^2^ = 0.9995). Dry extracts and α-AEF were weighed and dissolved in methanol resulting in concentrations of 10 mg/mL. Separations were performed for all extracts and α-AEF samples in triplicate.

### 4.8. Minimum Inhibitory Concentration and Minimum Bactericidal Concentration Determination

The antimicrobial activity of EOs, methanolic extracts, and α-AEF was investigated against reference human cocci pathogenic strains: *Staphylococcus aureus* MRSA (ATCC 43300), *S. aureus* MRSA (29213), *S. aureus* MSSA (ATCC 29213), *S. epidermidis* (ATCC 12228), *Enterococcus faecalis* (ATCC 29212), *E. faecalis* VRE (ATCC 51299), *E. faecium* (ATCC 19434) and *Micrococcus luteus* (ATCC 10240). All investigated strains were Gram-positive bacteria. Xanthohumol (Sigma-Aldrich Chemie GmbH, Vestenbersgreuth, Germany) reference compound was tested against *S. aureus* MRSA (ATCC 43300) in the concentration range from 2 to 1000 μg/mL.

All samples were screened in vitro for antibacterial activities using the broth microdilution method according to the European Committee on Antimicrobial Susceptibility Testing (EUCAST) [34] and Clinical and Laboratory Standards Institute guidelines [35] against the eight strains. These microorganisms came from the American Type Culture Collection (ATCC) and are routinely used for the evaluation of antibacterial compounds. 

The microbial cultures were first subcultured on nutrient agar at 35 °C for 18–24 h. Both Mueller–Hinton broth (MHB) and Mueller–Hinton agar (MHA) were used for the antimicrobial assay. Microbial suspensions of each strain were prepared in sterile saline (0.85% NaCl) with an optical density of the 0.5 McFarland standard scale, containing 1.5 × 10^8^ CFU/mL (Colony Forming Units/mL). Stock solutions were dissolved in dimethyl sulfoxide (DMSO) at a concentration of 200 mg/mL for the EOs and 50 mg/mL for the extracts. Subsequently, the minimum inhibitory concentration (MIC) of these samples was examined by the microdilution broth method, using two-fold dilutions in Mueller–Hinton broth prepared in 96-well polystyrene plates. The final concentrations of the studied EOs ranged from 0.015 to 32 mg/mL and from 0.001 to 2 mg/mL for extracts and α-AEF. To each well containing broth and serial dilutions of EOs or extracts with α-AEF, a bacterial suspension was added. After incubation, the MIC was assessed spectrophotometrically as the lowest concentration of the samples showing complete bacterial growth inhibition. Next, the minimum bactericidal concentration (MBC), defined as the lowest concentration of a compound which resulted in a >99.9% reduction in the CFU of the initial inoculum, was tested. The MBC was evaluated by removing the culture used for MIC determinations from each well and spotting onto Mueller–Hinton agar, and the plates were incubated under the appropriate conditions as before. The lowest compound concentration with no visible growth was considered to be the bactericidal concentration. All experiments were repeated and representative data were presented. Appropriate DMSO, antimicrobial compound (vancomycin) growth and sterile controls were carried out. The medium with no tested EOs or extracts with α-AEF was also used as a control. In this study, the MBC/MIC ratio was calculated in order to determine the bactericidal (MBC/MIC ≤ 4) or bacteriostatic (MBC/MIC > 4) effects of the EOs and extracts and their components [36,37,38]. In this study, no bioactivity was defined as an MIC > 1000 µg/ml, mild bioactivity as an MIC in the range 501–1000 µg/mL, moderate bioactivity with an MIC from 126 to 500 µg/mL, good bioactivity as an MIC in the range 26–125 µg/mL, strong bioactivity with an MIC between 10 and 25 µg/mL, and very strong bioactivity as an MIC < 10 µg/mL [37].

## 5. Conclusions

The most characteristic compounds for all investigated hop EOs were myrcene, α-humulene, (*E*)-β-caryophyllene and (*E*)-β-farnesene. Methanol extracts showed a high prevalence of α- and β-acids, prenylated flavonoids, especially xanthohumol and also some of the characteristic volatile compounds detected in the EOs. 

The highest anti-coccal activity was recorded for the methanol extract as well as for the EO of the Iunga hop variety. The activity of the hop extracts was at least one order of magnitude greater than those of the EOs. Very strong anti-coccal activity of the extract from the Iunga cultivar could be correlated with the highest concentration of xanthohumol in this extract. This compound showed high activity against *Staphylococcus aureus* MRSA (ATCC 43300), with an MIC value of 3.9 µg/mL. The most susceptible Gram-positive coccal strain to both EOs and extracts was *Micrococcus luteus* and the least susceptible was *Enterococcus faecium*. 

This work profiled the susceptibility of coccal human pathogens to different hop methanol extracts and EOs, showing that the antimicrobial properties of *H. lupulus* varieties are the result of complex phytochemistry.

These data suggest that extracts obtained from brewing quality hop species are valuable anti-Gram-positive agents and may be used as alternatives for treatment of infections caused by these bacteria. 

## Figures and Tables

**Table 1:** Essential oil yields, methanolic extraction efficiency and xanthohumol content in the investigated hops.

Hop Cultivar	Code	EO Content [%]	Extraction Efficiency [%]	Xanthohumol Concentration [%]
Iunga	In1	0.84	6.98	2.71
Marynka	Mr2	1.30	5.68	1.58
Sybilla	Sb3	1.08	5.32	1.80
Magnum	Mg4	1.50	6.92	0.39
Tradition	Tr5	0.60	6.28	1.07
Chinook	Ch6	0.68	5.50	1.20

**Table 2:** Results from the GC/MS analysis of hop essential oils. RI_exp._—retention index on ZB-5MS column, RI_lit._—retention index from the literature (MassFinder, NIST).

Compound Name	RI_exp_	RI_lit_	EO from Hop Samples—Relative Percentages					
			In1 *	Mr2	Sb3	Mg4	Tr5	Ch6
2-Methylpropyl 2-methylpropanoic acid ester	914	913	0.7	0.4	0.4	0.2	0.2	0.5
β-Pinene	980	978	0.6	0.6	0.6	0.6	0.4	0.6
Myrcene	990	987	27.6	28.7	36.1	37.0	23.8	24.1
Isobutyl isopentanoate	1002	1005	-	-	-	-	0.1	-
3-Methylbutyl isobutyrate	1012	1013	0.2	0.4	0.2	0.3	0.3	0.5
2-Methylbutyl isobutyrate	1015	1016	2.7	2.4	1.7	1.8	1.0	2.8
Methyl heptanoate	1023	1021	-	-	-	0.1	0.2	-
Limonene	1032	1025	0.2	0.2	0.2	0.2	0.1	0.2
β-Phellandrene	1034	1025	-	0.1	0.2	-	-	-
(*E*)-β-Ocimene	1048	1041	0.4	0.3	0.6	0.3	-	0.1
Methyl octanoate	1086	1061	0.2	0.1	0.1	0.4	0.2	0.7
2-Nonanone	1092	1090	0.1	0.2	-	-	0.1	-
Perillene	1100	1090	0.1	0.3	0.1	-	0.1	0.3
Linalool	1103	1101	0.4	0.7	0.4	0.5	1.0	0.7
Nonanal	1106	1102	0.1	-	-	-	0.2	0.3
Methyl nonanoate	1223	1227	-	-	-	-	0.3	-
Geraniol	1254	1235	0.6	0.1	-	-	0.4	-
Undecanone	1257	1273	0.2	-	-	0.2	0.1	0.2
2-Undecanone	1293	1291	0.8	0.7	0.4	0.6	1.2	0.5
Methyl 4-decenoate	1307	1303	1.3	1.2	1.1	1.7	1.5	2.3
Methylgeranate	1322	1326	0.5	2.3	1.0	0.4	0.3	1.0
α-Ylangene	1378	1376	0.2	0.2	0.1	0.2	0.2	0.6
α-Copaene	1384	1379	0.5	0.3	0.3	0.5	0.4	0.5
2-Dodecanone	1395	1381	-	-	0.2	0.2	0.2	-
(*E*)-β-Caryophyllene	1432	1421	10.2	7.3	9.8	8.2	10.2	6.1
(*E*)-α-Bergamotene	1441	1434	0.4	1.3	0.7	0.4	0.5	1.0
(*E*)-β-Farnesene	1454	1446	0.9	18.8	8.1	1.1	1.5	3.4
α-Humulene	1469	1455	30.4	14.1	23.9	27.2	33.1	15.7
Germacrene D	1484	1479	1.3	1.0	1.1	0.8	1.3	2.0
Sesquiterpene, m ** = 204, bp = 105	1488		-	-	-	-	1.2	-
Muurola-4,9-diene	1489	1490	0.1	-	-	0.1	0.1	-
2-Tridecanone	1496	1494	0.5	0.3	0.3	0.5	1.1	0.6
Zingiberene	1499	1496	-	-	-	-	-	3.5
β-Selinene	1502	1497	0.7	0.7	0.6	0.4	0.6	1.9
(*E*,*E*)-β-Farnesene	1506	1498	1.8	1.8	1.6	3.6	0.8	1.8
α-Selinene	1509	1507	-	-	-	-	0.9	2.8
γ-Cadinene	1513	1520	0.2	0.3	0.2	-	0.2	0.6
Sesquiterpene, m = 204, bp = 161	1524		1.0	0.6	0.8	0.8	1.1	1.4
β-Cadinene	1527	1526	1.5	0.9	1.2	1.2	1.6	1.9
Sesquiterpene, m = 204, bp = 159	1533		0.4	0.3	0.3	0.2	0.4	0.7
Sesquiterpene, m = 204, bp = 105	1548		0.2	0.2	0.2	0.2	0.3	0.3
Sesquiterpene, m = 204, bp = 161	1551		-	-	-	-	-	0.6
Selina-3,7(11)-diene	1554	1542	0.1	-	-	-	0.1	0.7
Caryophyllene oxide	1598	1578	0.6	0.9	0.4	0.2	0.9	0.8
α-Humulene epoxide I	1615	1593	0.2	0.2	-	-	0.4	0.3
α-Humulene epoxide II	1628	1602	1.5	1.4	0.6	0.5	2.3	1.8
α-Humulene epoxide III	1651	1626	0.4	0.4	0.2	0.2	0.6	0.6
Sesquiterpene, m = 204, bp = 161	1657		0.3	0.3	0.2	0.2	0.4	0.5
Sesquiterpene, m = 204, bp = 43	1673		0.4	0.3	0.2	0.3	0.6	0.8
Sesquiterpene, m = 204, bp = 43	1677		-	-	-	-	0.3	-
Longifolene aldehyde	1688	1668	0.1	-	-	0.1	0.2	-
TOTAL			90.6	90.3	93.9	91.4	92.9	85.3
(*E*)-β-Caryophyllene + α-Humulene			40.6	21.4	33.7	35.4	43.3	21.8

* For codes see Table 1. ** m—mass peak, bp—base peak.

**Table 3:** The most abundant volatile components identified in methanolic extracts. RI_exp._—retention index on ZB-5MS column, RI_lit._—retention index from the literature (MassFinder, NIST).

Compound Name	RI_exp_	RI_lit_	Fragmentation Ions	In1 ^#^	Mr2	Sb3	Mg4	Tr5	Ch6
(*E*)-β-Caryophyllene	1423	1421	*204* *-189-161-147-133-**93** **-69-41	1.2	1.4	1.3	1.4	-	-
(*E*)-β-Farnesene	1452	1446	*204*-161-133-93-**69**-41	-	5.0	1.4	-	-	-
α-Humulene	1460	1455	*204*-147-121-**93**-80-41	4.0	2.9	3.5	5.0	3.3	1.8
Colupulone	2387	-	*400*-331-289-**275**-233-221-205-177-109-**69**-41	17.4	22.3	22.0	12.0	14.7	17.7
Adlupulone	2455	-	*414*-345-**289**-277-233-221-205-177-135-109-**69**-41	4.1	3.3	4.3	2.1	3.1	3.4
Lupulone	2463	2482	*414*-345-**289**-277-235-205-177-109-**69**-41	10.1	13.1	18.5	7.9	12.5	14.3

* parent ion mass in italics; ** high-intensity peaks in bold. ^#^ relative percentages of volatiles present in methanolic extracts, for codes see Table 1.

**Table 4:** The most abundant compounds detected in the hop methanolic extracts by LC/MS (negative ion mode).

RT	Compound	Molecular Ion [M-H]^-^	Fragmentation Ions	In1 *	Mr2	Sb3	Mg4	Tr5	Ch6
34.23	8-Prenylnaringenin	339.1310	219.0701; 119.0518	3.8	1.6	13.1	3.2	5.8	3.8
36.65	Naringenin glucuronide	447.2199	383.2218; 271.0976	41.6	10.3	33.5	40.4	12.1	6.7
37.79	Xanthohumol	353.1350	233.0791; 119.0483	37.5	22.3	33.6	13.3	6.7	2.8
43.68	Cohumulone	347.1968	278.1251; 235.0701; 207.0733	34.5	tr	36.4	24.0	30.0	23.2
45.75	Humulone	361.2131	292.1428; 249.0877; 221.0904	50.8	tr	2.4	37.1	37.1	35.2
46.56	Adhumulone	361.2158	292.1432; 249.0882; 221.0907	24.4	tr	18.5	8.6	7.2	3.0
47.33	Unidentified flavonoid	443.2972	259.1088	31.2	tr	42.3	15.5	20.3	7.9

* relative abundance of compounds present in methanolic extracts, for codes see Table 1; tr—traces.

**Table 5:** The activity data of the essential oils expressed as MIC, MBC [mg/mL] and MBC/MIC ratio values against the reference strains of cocci from Gram-positive bacteria. For codes of hop cultivars see Table 1.

Essential Oils	The Reference Strains of Bacteria							
	*Staphylococcus aureus* ATCC 43300 (MRSA)	*Staphylococcus aureus* ATCC BAA-1707 (MRSA)	*Staphylococcus aureus* ATCC 29213 (MSSA)	*Staphylococcus epidermidis* ATCC 12228	*Micrococcus luteus* ATCC 10240	*Enterococcus faecalis* ATCC 51299 (VRE)	*Enterococcus faecalis* ATCC 29212	*Enterococcus faecium* ATCC 19434
Minimum Inhibitory Concentration [mg/mL]								
In1	4	8	8	4	1	4	8	8
Mr2	8	16	8	4	4	8	16	16
Sb3	8	16	16	4	8	8	16	16
Mg4	8	16	16	8	8	8	16	16
Tr5	4	8	4	2	2	4	16	16
Ch6	4	8	4	4	1	8	16	16
Minimum Bactericidal Concentration [mg/mL]								
In1	4	8	8	16	4	32	32	>32
Mr2	8	16	16	8	8	32	32	32
Sb3	16	16	16	16	16	32	32	32
Mg4	16	16	16	32	8	>32	>32	>32
Tr5	16	8	8	8	4	16	32	32
Ch6	8	8	8	8	2	16	32	32
MBC/MIC								
In1	1	1	1	4	4	8	4	>4
Mr2	1	1	2	2	2	4	2	2
Sb3	2	1	1	4	2	4	2	2
Mg4	2	1	1	4	1	>4	>2	>2
Tr5	4	1	2	4	2	4	2	2
Ch6	2	1	2	2	2	2	2	2

**Table 6:** The activity data of the methanol extracts expressed as MIC, MBC [µg/mL] and MBC/MIC ratio values against the reference strains of cocci from Gram-positive bacteria. For codes of hop cultivars see Table 1.

Extracts	The Reference Strains of Bacteria							
	*Staphylococcus aureus* ATCC 43300 (MRSA)	*Staphylococcus aureus* ATCC BAA-1707 (MRSA)	*Staphylococcus aureus* ATCC 29213 (MSSA)	*Staphylococcus epidermidis* ATCC 12228	*Micrococcus luteus* ATCC 10240	*Enterococcus faecalis* ATCC 51299 (VRE)	*Enterococcus faecalis* ATCC 29212	*Enterococcus faecium* ATCC 19434
Minimum Inhibitory Concentration [µg/mL]								
In1	7.8	7.8	7.8	15.6	7.8	31.3	7.8	7.8
Mr2	15.6	15.6	31.3	31.3	15.6	31.3	15.6	31.3
Sb3	7.8	7.8	15.6	31.3	7.8	15.6	15.6	31.3
Mg4	31.3	31.3	31.3	31.3	31.3	31.3	31.3	62.5
Tr5	7.8	15.6	15.6	31.3	7.8	31.3	15.6	31.3
Ch6	15.6	15.6	15.6	62.5	15.6	31.3	31.3	62.5
α-AEF *	62.5	31.3	15.6	31.3	15.6	31.3	62.5	31.3
Xanthohumol	3.9	nd **	nd	nd	nd	nd	nd	nd
Minimum Bactericidal Concentration [µg/mL]								
In1	7.8	62.5	15.6	15.6	15.6	250	125	2000
Mr2	15.6	125	31.3	62.5	31.3	1000	1000	2000
Sb3	7.8	62.5	31.3	31.3	31.3	1000	500	2000
Mg4	62.5	125	500	125	31.3	>2000	1000	>2000
Tr5	15.6	62.5	31.3	31.3	62.5	1000	1000	2000
Ch6	15.6	125	500	62.5	15.6	2000	1000	2000
α-AEF	62.5	500	250	125	15.6	2000	1000	2000
Xanthohumol	250	nd	nd	nd	nd	nd	nd	nd
MBC/MIC								
In1	1	8	2	1	2	8	16	256
Mr2	1	8	1	2	2	32	64	64
Sb3	1	8	2	1	4	64	32	64
Mg4	2	4	16	4	1	>64	32	>64
Tr5	2	4	2	1	8	32	64	64
Ch6	1	8	32	1	1	64	32	32
α-AEF	1	16	16	4	1	64	16	64
Xanthohumol	64	nd	nd	nd	nd	nd	nd	nd

* α-acid-enriched fraction. ** nd—not determined.

## Data Availability

Extended data are available from the first author.
